# Funding global health product R&D: the Portfolio-To-Impact Model (P2I), a new tool for modelling the impact of different research portfolios

**DOI:** 10.12688/gatesopenres.12816.2

**Published:** 2018-07-19

**Authors:** Robert F Terry, Gavin Yamey, Ryoko Miyazaki-Krause, Alexander Gunn, John C. Reeder

**Affiliations:** 1 The Special Programme for Research and Training in Tropical Diseases, World Health Organization, Geneva, 1211, Switzerland; 2 Center for Policy Impact in Global Health, Duke Global Health Institute, Durham, NC, 27710, USA

**Keywords:** research and development, innovation, neglected diseases, global health

## Abstract

**Background: **The Portfolio-To-Impact (P2I) Model is a novel tool, developed to estimate minimum funding needs to accelerate health product development from late stage preclinical study to phase III clinical trials, and to visualize potential product launches over time.

**Methods: **A mixed methods approach was used. Assumptions on development costs at each phase were based on clinical trial costs from Parexel’s R&D cost sourcebook. These were further refined and validated by interviews, with a wide variety of stakeholders from Product Development Partnerships, biopharmaceutical and diagnostic companies, and major funders of global health R&D.

**Results**: the tool was used to create scenarios describing the impact, in terms of products developed, of different product portfolios with funding ranging from $1 million per annum through to $500 million per annum. These scenarios for a new global financing mechanism have been previously presented in a report setting out the potential for a new fund for research and development which would assist in accelerating product development for the diseases of poverty.

**Conclusion:** The P2I tool does enable a user to model different scenarios in terms of cost and number of health products launched when applied to a portfolio of health products.  The model is published as open access accompanied with a user guide.  The design allows it to be adapted and used for other health R&D portfolio analysis as described in an accompanying publication focussing on the pipeline for neglected diseases in 2017. We aim to continually refine and improve the model and we ask users to provide us with their own inputs that can help us update key parameters and assumptions.  We hope to catalyse users to adapt the model in ways that can increase its value, accuracy, and applications.

## Introduction

In 2012 the World Health Organization (WHO) considered the findings of a report from an expert working group into research and development to meet the health needs of developing countries. Among a number of recommendations this highlighted the need for better financial incentive mechanisms for health product research and development (R&D) for the neglected diseases. These diseases often have the greatest burden among the poorest populations and this absence of purchasing power– described as market failure – means there is often a need for R&D to be financed from public or philanthropic sources, rather than relying on conventional market forces ^1^.

As a consequence, in 2014, the Special Programme for Research and Training in Tropical Diseases (TDR) was asked by the World Health Organization to explore potential financial mechanisms – in particular a new fund for R&D - which would assist in accelerating product development for the diseases of poverty, including Type III and II diseases as well as the specific R&D needs of developing countries in relation to Type I diseases ^2^.

TDR, the Special Programme for Research and Training in Tropical Diseases, is a global programme of scientific collaboration that helps facilitate, support and influence efforts to combat diseases of poverty. It is hosted at the World Health Organization (WHO), and is sponsored by the United Nations Children’s Fund (UNICEF), the United Nations Development Programme (UNDP), the World Bank and WHO. Established in 1975 TDR has co-developed 12 new drugs for tropical parasitic diseases, building research capacity in three generations of public health leaders throughout the developing world and pioneered the role of communities and community health workers in delivering health interventions in many low-income countries ^3^.

The disease Types I, II and III were first introduced by the Commission on Macroeconomics and Health and elaborated in the report of the Commission on Intellectual Property Rights, Innovation and Public Health. The definition of diseases into Types mixes a number of concepts together including the wealth of a country between rich and poor; the state of its development between developed and developing, and most importantly a measure of the burden of diseases by the incidence of the disease within the population. The definitions themselves are combined such that ^4^:

**Type I** diseases: are incident in both rich and poor countries, with large numbers of vulnerable populations in each and the focus is on the R&D needs specific to developing countries.
**Type II** diseases: are incident in both rich and poor countries, but with a substantial proportion of the cases in poor countries.
**Type III** diseases: are those that are overwhelmingly or exclusively incident in developing countries.

In response to this request, TDR conducted a study to analyse current funding landscape and product pipelines of Type III and II diseases and to identify funding bottlenecks and operational issues. The study included the development of a financial model to create scenarios that could estimate the impact of different sized portfolios of R&D products. The findings of the study were published as a report entitled Health Product Research & Development Fund: A Proposal for Financing and Operation (2016) – referred to in this article as TDR Report 2016 ^5^.

Here, we describe in more detail the creation of the financial tool called the Portfolio To-Impact (P2I) model, a novel financial forecasting tool to estimate funding needs of pharmaceutical product development over the period 2017–2030. In this modelling outputs are relative to the number of projects that can be funded per year and subsequently year-on-year. The original 13 year period was the time period chosen at that time for the purpose of demonstrating to the Member States of WHO the different impact of global R&D funds of different sizes with a realistic potential for producing product launches.

## Methods

**Figure 1.**
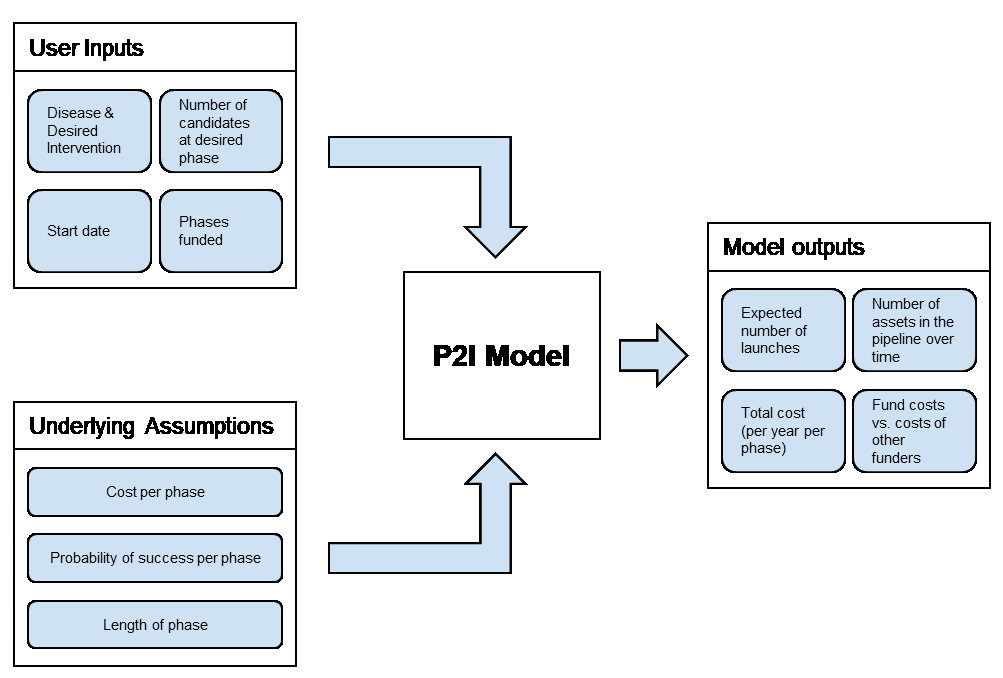
Conceptual overview of the P2I financial portfolio model.

The P2I model is a financial portfolio tool that enables users to estimate funding needs to move candidate health products through the pipeline from late stage preclinical to phase III clinical trials, as well as potential product launches over time. For the purposes of this paper we modelled outcomes from a starting point of 2017 up to 2030. The modelling tool, which is deterministic, can estimate the costs of delivering different portfolios of product interventions and the health impacts of these portfolios. It is based on assumptions for costs, attrition rates, and cycle times for four development phases (preclinical to phase III) for eleven different kinds of medical products, called *archetypes*. A conceptual overview of the model is given in Figure 1.

This section describes: (a) how the archetypes were determined; (b) the development of the model’s assumptions; (c) the health impact methodology; (d) the construction of the P2I Microsoft Excel tool; and (e) estimating capacity constraints – the number of projects that can be supported per annum.

### a) Determination of archetypes

Medical products were classified into eleven different types of intervention. Table 1 shows the basis for this classification and examples of each archetype. Products were first classified into five major categories: new vaccine, new chemical entity (NCE), repurposed drug, new biologic, or diagnostic. Each of these categories was then further sub-divided; for example, new vaccines were sub-divided into simple versus complex. The main differentiation point for archetypes within each of these five broad categories (e.g., within the new vaccine category) is whether the approach or mechanism of action is novel or has already been validated. For each of the 11 archetypes, separate assumptions on costs, attrition rates, and cycle times per phase were developed to recognize the variation in R&D characteristics and more accurately estimate R&D costs.

**Table 1.** Intervention archetypes.

Archetype		Description	Examples
Vaccine	Simple	Platform has been used to develop other vaccines	Hepatitis A, Hepatitis B, Polio
	Complex	Requires completely novel approach; no platform; no existing research	Pneumococcal conjugate vaccine (PCV), Meningitis B
New Chemical Entity (NCE)	Simple	Validated target or mechanism of action	Primaquine
	Innovative	Novel target or mechanism of action with understanding of disease pathogenesis	Ibrutinib
	Complex	Novel target or mechanism of action without understanding of disease pathogenesis	Imatinib
Repurposed Drug	Simple	Drug has sufficient safety data to start development in Phase II	Azithromycin, Doxycylcine
	Complex	Drug requires some Phase I clinical trials to verify safety in humans	Moxidectin
Biologic	Simple	Validated target or mechanism of action	IL-17 antibody
	Complex	Novel target or mechanism of action	Natalizumab
Diagnostics	Assay development	Development of a diagnostic assay	Lateral flow tests, Quantitative molecular tests
	Simple technical platform development	Development of a technological platform that enhances current technology	Hypersensitive malaria rapid diagnostic test (RDT)

### b) Development of assumptions

The assumptions on development costs at each phase were initially based on a bottom-up analysis of clinical trial costs from Parexel’s R&D cost sourcebook, which is the leading resource for statistics, trends, and proprietary market intelligence and analyses within the biopharmaceutical industry ^6^. These were then further refined and validated following interviews, with a wide variety of stakeholders from PDPs, biopharmaceutical and diagnostic companies, and major funders of global health R&D.

Interviews were semi-structured held over the phone between the researchers creating the models and senior R&D stakeholders from identified entities (question used in the interviews are presented in Supplementary File 1). Conversations were confidential to protect private research data of entities involved, hence no digital recordings were created. We used these interviews to review and validate the cost assumptions from the R&D cost sourcebook.

The assumptions on attrition rates and cycle times at each phase were initially based on a review of over 25,000 development candidates for attrition rates and cycle time ^7^. These assumptions were further refined and validated based on academic literature ^8^,^9^, industry publications ^6^,^7^, and stakeholder interviews. Stakeholders were initially identified from across the networks of the authors and stakeholders were invited to recommend further interviewees as appropriate.

**Figure 2.**
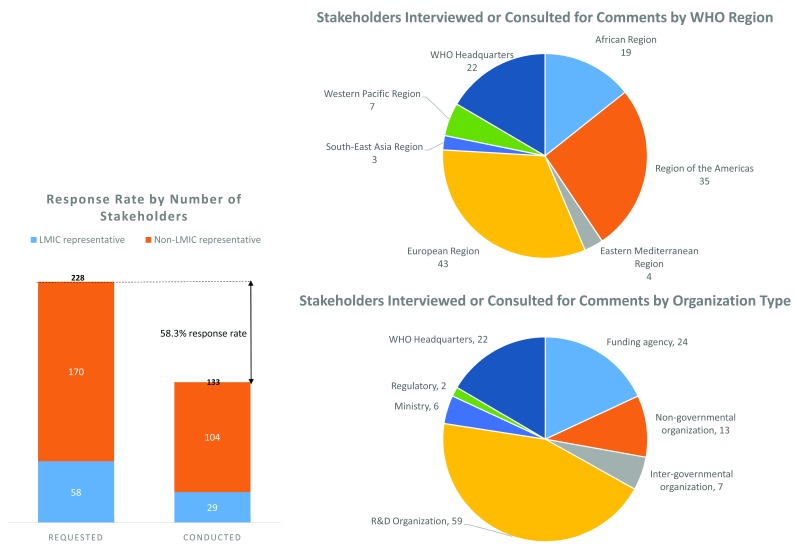
Stakeholder interviews—response rate and stakeholder categories.

For the stakeholder interviews, a total of 228 stakeholders representing a cross-section of the global R&D landscape were contacted to request an interview and 133 agreed to be interviewed, a response rate of 58%. Of these 113 stakeholders who agreed, 29 were representatives of low- and middle-income countries (LMICs). The breakdown of these stakeholders by WHO region and type of organization is shown in Figure 2. Supplementary File 2 shows the full list of stakeholder organizations contacted and which organizations agreed to be interviewed. The interviews were conducted to gather external perspectives, to help validate emerging assumptions and findings, and to gain stakeholder feedback on the seven different pooled fund scenarios we generated using the P2I model (described further below).

As a final validation step, the P2I model and its assumptions were reviewed by TDR’s Scientific and Technical Advisory Committee, who provided an additional round of expert inputs.

***Development of assumptions on costs per phase.*** The R&D scope of the model begins at the preclinical phase (after lead optimization) and ends at phase III clinical trials. In the P2I model, the estimated R&D costs include direct expenses, such as investigator grants and clinical supplies, internal headcount costs (full time equivalents [FTEs]), and in-kind (non-monetary) contributions that partnership-based organizations, such as PDPs, may receive from industry partners (e.g., lab costs, some internal headcount). The included costs are shown in Table 2.

**Table 2.** Categories of costs included in the P2I model.

Cost category	Included costs
**Preclinical costs ^a^**	
**Patient-driven costs (Phases I-III)**	Number of patients
	PI grant per patient
	Clinical supplies
	Monitoring
	Laboratory tests
	Data management
**Site costs** **(Phases I-III)**	Site start-up cost
	Number of sites
**Internal FTE costs (Phases I-III)**	Clinical operations FTE
	Contracting and legal FTE
	Data management and statistics FTE
	Global clinical trial management FTE
	Global medical and regulatory affairs FTE
	Regional clinical trial management FTE
	Regional medical and regulatory affairs FTE
**Overarching costs (Phases I-III)**	Phase I biomarker costs

Derived from Liao XC ^9^. PI: principal investigator; FTE: full time equivalent

Several costs are *not* included in the P2I model as they were deemed out of scope. These include: all costs related to basic research through lead optimization; chemistry, manufacturing, and controls (CMC); good manufacturing practice (GMP); manufacturing build up and scale-up costs; regulatory or registration fees (post-phase III); and all post-market commitments (e.g., phase IV pharmacovigilance studies).

R&D costs estimates were developed for preclinical through phase III and include lower bound, upper bound, and point estimates ( Table 3). As mentioned, the cost estimates were initially derived from a bottom-up analysis using Parexel’s R&D cost sourcebook ^6^ and subsequently validated by interviews with stakeholders from the pharmaceutical industry and PDPs and through triangulation with academic and industry literature ^6^,^9^. These development cost estimates reflect an R&D system that would be performing in a highly cost efficient manner. The cost estimates do not include capability building or infrastructure development expenses and thus reflect an idealized model in which R&D is performed in an established and streamlined organization, facility or partnership. The detailed cost assumptions separately for vaccines, NCEs, repurposed drugs, and biologics is shown in Supplementary File 3.

**Table 3.** Development cost assumptions per phase per archetype.

Archetype		Cost estimates per phase ($, Millions)							
		Preclinical		Phase 1		Phase 2		Phase 3	
		Lower bound, Upper bound	Point estimate	Lower bound, Upper bound	Point estimate	Lower bound, Upper bound	Point estimate	Lower bound, Upper bound	Point estimate
Vaccine	Simple	3.3, 10.0	6.7	1.8, 2.7	2.2	7.4, 19.0	13.2	56.6, 165.6	111.1
	Complex	8.3, 24.9	16.6	1.9, 3.0	2.5	7.8, 20.0	13.9	67.9, 198.7	133.3
New Chemical Entity (NCE)	Simple	2.5, 7.5	5.0	1.8, 2.7	2.2	3.7, 7.9	5.8	11.5, 54.1	32.8
	Innovative	5.0, 10.0	7.5	4.4, 5.3	4.8	3.9, 8.3	6.1	12.1, 55.4	34.5
	Complex	7.5, 12.5	10.0	6.9, 7.9	7.4	4.1, 8.7	6.4	12.6, 59.6	36.1
Repurposed Drug	Simple	N/A	N/A	N/A	N/A	3.7, 7.9	5.8	10.0, 25.2	17.6
	Complex	2.5, 7.5	5.0	1.7, 2.7	2.2	3.7, 7.9	5.8	10.0, 25.2	17.6
Biologic	Simple	5.4, 16.2	10.8	1.9, 3.0	2.4	4.5, 10.5	7.5	27.7, 80.5	54.1
	Complex	16.2, 27.0	21.6	7.0, 8.3	7.6	5.0, 11.6	8.3	30.5, 88.5	59.5
		Selection and validation of markers		Development		Regulated trials beyond EUO/CE			
Diagnostics	Assay development	1.0, 5.0	3.0	1.0, 3.0	2.0	1.0, 6.0	3.5		
	Simple technical platform development	N/A	N/A	50.0, 150.0	100.0	1.0, 6.0	3.5		

***Development of assumptions on attrition rates and cycle times per phase.*** Probability of success assumptions and cycle time per phase assumptions ( Table 4) were similarly developed for each of the eleven archetypes from preclinical through phase III (or the relevant phases for diagnostics). For both sets of assumptions data was sourced from PDP stakeholder interviews, experts in the pharmaceutical industry, published literature, and industry databases ^6^,^7^.

**Table 4.** Probability of success (attrition rate) and cycle time (length of phase) assumptions per phase per archetype.

Archetype		Length of phase (years)				Probability of success (%)			
		Preclinical	Phase 1	Phase 2	Phase 3	Preclinical	Phase 1	Phase 2	Phase 3
Vaccine	Simple	3.36	1.57	2.23	2.33	41.0	68.4	45.9	70.8
	Complex	3.33	1.97	3.71	3.50	41.0	50.0	21.6	63.6
New Chemical Entity (NCE)	Simple	2.49	1.80	3.38	3.18	65.0	59.7	38.8	69.1
	Innovative	2.70	1.81	3.35	3.10	60.0	51.9	28.4	57.8
	Complex	2.87	1.93	3.51	2.80	55.0	57.2	19.7	40.3
Repurposed Drug	Simple	0.00	0.00	2.14	2.14	100.0	100.0	45.7	68.1
	Complex	2.33	1.63	2.14	2.14	75.0	58.5	45.7	68.1
Biologic	Simple	3.29	1.62	2.47	2.10	75.0	66.2	44.3	70.9
	Complex	3.24	1.49	4.16	3.38	77.0	69.6	32.2	62.5
Diagnostics	Assay development	1.00	1.25	1.33	0.00	50.0	100.0	100.0	100.0
Diagnostics	Simple Technical Platform Development	0.00	2.50	2.00	0.00	100.0	75.0	100.0	100.0

The assumptions were based on the Pharmaprojects database, a subscription database that tracks R&D filings for over 60,000 individual assets ^7^. Specific sub-selections of the assets were made to differentiate between archetypes. Candidates that reported R&D activity from 2007–2014 and met specific criteria for each archetype were included in the analysis. After review with relevant stakeholders and other R&D experts, the assumptions were adjusted (primarily for the preclinical phase) using academic literature. The methodology used to identify appropriate candidates and the sample size is shown in Table 5, and the model assumptions on development costs, attrition rates, and cycle times for each phase were based on data on over 25,000 product candidates.

**Table 5.** Probability of success (attrition rate) and cycle time methodology.

Archetype (N = number of product candidates used in estimation)	Functional definition for estimating attrition rate and cycle time ^a^^b^	Other adjustments made to assumptions
Simple vaccine **(N=247)**	All vaccines listed for indications requiring a simple vaccine based on McKinsey pharmaceutical practice classification; excludes influenza vaccines	Adjusted preclinical phase probability as per Pronker *et al.*, 2013 ^12^
Complex vaccine **(N=409)**	All vaccines listed for indications requiring a complex vaccine based on McKinsey pharmaceutical practice classification	
Simple NCE **(N=3655)**	All NCEs with more than 1 candidates in Phase III or higher as proxy for validated target	Adjusted preclinical phase probability of success to 55–65% per McKinsey pharmaceutical practice
Complex NCE **(N=4426)**	All NCEs with 1 or 0 candidates in Phase III or higher as proxy for non-validated target	
Innovative NCE **(N=14425)**	All NCEs excluding reformulations	N/A
Simple drug repurposing **(N=3768)**	All reformulations	N/A
Complex drug repurposing **(N=3768)**	All reformulations	Adjusted preclinical phase probability of success to 75% per Biovista Inc. Drug Repositioning Factsheet
Simple biologic **(N=4247)**	All other biologics not categorized as complex	Adjusted preclinical phase probability of success to 76% per KMR Pharmaceutical Benchmarking Forum for large new molecular entities; Assumed same spread between simple and complex probability of success from Pharmaprojects database
Complex biologic **(N=1440)**	All biologics for pharmacology classes categorized as complex, including gene therapy, antisense therapy, gene delivery vector, RNA interference, stem cell therapy, cellular therapy, and lytic virus	

All assumptions based on Pharmaprojects database (>60,000 individual assets captured) and McKinsey Attrition Analytics Toolkit unless otherwise noted. All data points are from 2007 to 2014.

Due to the lack of academic literature available on development characteristics for diagnostics, the archetype definitions and assumptions were based on expert interviews with a leading PDP specializing in diagnostics and with diagnostics developers from large pharmaceutical companies.

### c) Health impact methodology

The P2I model allows users to estimate the impact of a launched product on both disability, measured in disability-adjusted life years (DALYs) averted, and mortality, measured in deaths averted. The economic value of the DALYs averted is also calculated in terms of US dollars. The user is expected to make an informed estimate of the expected reduction in disease burden and expected reduction in mortality based on project-specific characteristics and estimates. These characteristics/estimates include, but are not limited to, the impact of improved efficacy over standard of care on mortality and morbidity, coverage rates, and disease prevalence and incidence.

The health impact calculation determines the DALYs averted, associated economic value, and deaths averted for a single launched intervention, irrespective of product archetype. The 2012 disease burden (DALYs) and mortality data for each Type III and II diseases were based on the WHO Global Health Estimates ^10^. The DALYs averted metric is calculated by multiplying the 2012 LMIC disease burden by the expected reduction in disease burden (determined on a case-by-case basis by the model user). The same methodology is used for the deaths averted metric based on the users’ input.

The associated economic value of the health impact is based on a valuation of $500 per DALY averted, as used in estimates by the International Finance Facility for Immunization (IFFIm) ^11^. The IFFIm recognizes that the true economic value of a single DALY averted is “likely much higher,” implying that the economic value is a relatively conservative estimate of the actual economic impact. The intention is not to arrive at a ‘true’ economic estimate. The use of such an estimate is to inform priority setting through the comparison of the impact of one product versus another. Further details of the health impact methodology are shown in Supplementary File 4.

### d) Construction of the Microsoft Excel tool

The assumptions on costs, attrition rates, and cycle time per phase for the 11 different archetypes and the health impact estimation formulae were used to create a P2I tool in Microsoft Excel 2016 ( Supplementary File 5). A users’ guide to this tool is presented in Supplementary File 6. In brief, the tool allows users to input a portfolio of up to 150 product development projects. Users can select which diseases these projects will target and which product archetypes will be modelled. They can then choose a specific number of compounds that will reach a specific phase, which can either be *outcome-based* (e.g. “I want three compounds in Phase III” or “I want one launch”) or *funding-based* “(I want to fund two phase I projects”). Users select the year that each project for each archetype would start and the development phase at which the modelling should begin. The model also gives users the ability to set which phases their particular funding organization would fund, and to estimate health impacts of product launches. It is worth noting here that the model calculates the expected number of candidates moving from one phase to the next, including the expected launches. This means that on average, such a portfolio is expected to yield a certain number of launches; this is a floating-point number in the model. As an actual launch is binary the number of expected launches should then be rounded for presentation purposes.

### e) Estimating capacity constraints – the number of projects that can be supported per annum.

There is a final parameter which allows the user to model the impact of the capacity of an R&D system to absorb new projects on an annual basis. For a pooled fund operating at a global level this is set to 100% for each project i.e. it assumes everything that could be funded could be moved through the pipeline. Within this model changes to constrain the annual capacity parameter need to be applied per project/row in the Excel model sheet in line with the User Guide. Within the TDR Report 2016 the range of capacity constraints and portfolio considerations (for example a strategy of “quick wins” focused on mainly re-purposed drugs) were developed by the team. This allowed us to explore the relationship between the total product pipeline at the start of the funding process, the number of new projects supported per year, the number of launches in 2030 and funding requirements. In order to derive final recommendations in the TDR Report 2016 a range of funding scenarios using different strategies (e.g. “quick wins” vs. a mixed model of some repurposed and some new chemical entities) were illustrated in the report.

## Results

The P2I tool was used to develop a series of scenarios used to estimate the impact of launching a new fund for global health R&D, of varying fund sizes. The funds would support the launch of new health products in 2030 if they became operational in 2017. The results are previously reported in full in the TDR Report 2016.

A number of scenarios were modelled and discussed with R&D stakeholders with respect to how each fund could contribute to a new financial mechanisms to stimulate global health R&D ( Supplementary File 2 lists the stakeholders). These interviews revealed a broad spectrum of options for the financial mechanism, ranging from a specialized group that sets and communicates priorities, to a large global fund with its own secretariat. The seven scenarios that we modelled were chosen based on their feasibility and stakeholders’ willingness to implement them ( Table 6).

**Table 6.** Overview of seven funding scenarios.

Scenario Number	Annual fund size
1	up to US$ 1m to support passive coordination of R&D
2	up to US$ 5m to support prioritization of R&D
3	a small fund of approx. US$ 15m
4	a PDP-sized fund of approx. US$ 50m
5	a medium sized fund of approx. US$ 100m
6	a large fund of approx. US$ 300m
7	a global fund of approx. US$ 500m

**Figure 3.**
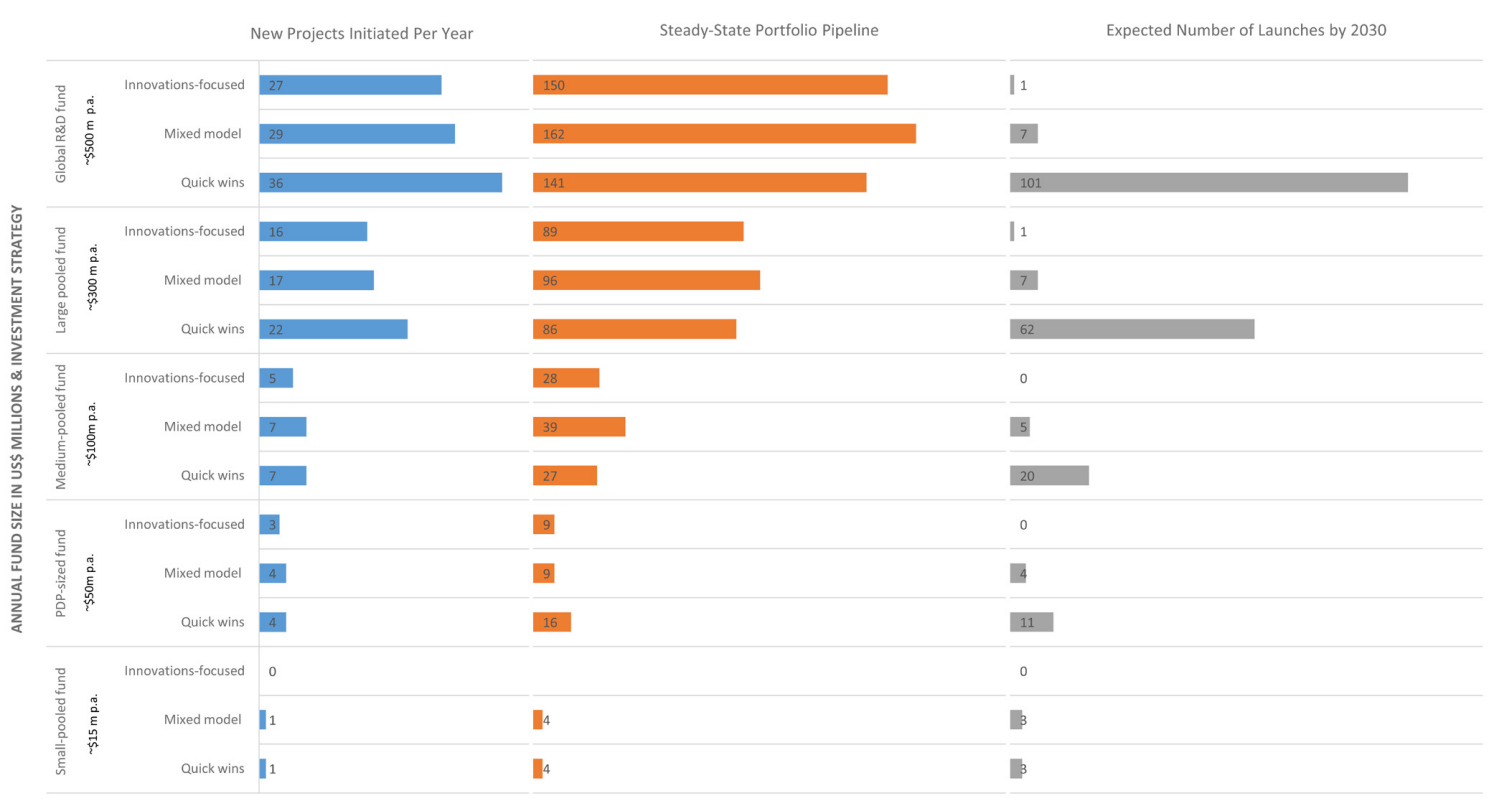
Spectrum of financial mechanisms explored.

Scenarios 1 and 2 describe the funds required for different degrees of priority setting and coordination of R&D at the global level. In scenarios 3 – 7 we used the P2I model to evaluate each of the five fund options that would support R&D projects by estimating the expected impact of various investment portfolios. The analysis evaluates the full development costs from preclinical (after lead optimization phase) through to phase III, accounting also for in-kind contributions from industry partners. Recognizing the numerous permutations of potential funding focus areas, a spectrum of financing focus strategies was constructed and explored for each of the fund sizes ranging from a fund of $15 million per annum up to $500 million per annum. This assumed three strategies. One focussed on quick wins and repurposing of existing drugs, one focussing on new innovations and a mixed model where the portfolio is balanced equally between these two approaches. The number of new projects that would need to be initiated each year (assuming attrition in existing projects), what the number of projects would be in a steady state portfolio, how many health products might be expected to be launched are shown in Figure 3.

## Discussion

We developed a flexible, portfolio-based financial model, the P2I model, which can analyse and estimate the financing needs of various R&D investment portfolios for poverty-related and neglected diseases (PRNDs). The model can estimate preclinical to launch costs of a portfolio of candidates of 11 different archetypes: new vaccines (simple, complex); NCEs (simple, innovative, and complex); repurposed drugs (simple, complex); new biologics (simple, complex); and diagnostics (assay development, simple technology platform development). It also estimates the outputs (i.e. number of launches) that can be expected from investing in a specific portfolio of candidates and the timeframe for these launches. Finally, a constraint parameter can be used to model the number of projects that can be undertaken within an R&D system. R&D funders can therefore use the P2I model to improve their understanding of which health products they could potentially launch for their specific investments. To the best of our knowledge, the P2I model is the first model that allows users to estimate the costs and outputs across a whole *portfolio* of candidates and, as such, it can help to guide overall portfolio management by informing priority setting.

The P2I model is not a tool for estimating the exact costs of developing a specific product or for estimating the timing or probability of success of individual projects; instead, it estimates costs of different portfolios selected by the user based on aggregates of historical data. The model can help to show R&D funders *where across the portfolio of candidates* they should focus their investments to maximize the chances of getting the most meaningful outputs (e.g., a new chemical entity or vaccine for a disease that is missing such tools). By showing the costs and outputs of different scenarios, it can help to guide R&D investments. By estimating the likely impacts of a product in reducing disability and mortality, funders can use the P2I model to forecast the different trade-offs from different investments. The tool can also be used for financial planning—for example, it can help to determine the number of projects requiring oversight per year to inform staffing needs.

We used the model to forecast the potential impacts of a novel pooled fund for R&D of various sizes with a focus on neglected diseases, these findings have been previously published in the TDR Report 2016 ^5^. Seven different scenarios were modelled, based on different annual fund sizes, ranging from US$1 million to help improve R&D coordination through to a global fund of over $500 million that could fund 140–160 projects across a range of priority areas. The study found that an annual disbursement of at least $100 million would be required (i.e. a medium sized fund) to fill significant gaps in the R&D pipeline. A fund of this size could support 25–40 projects. If it became operational in 2017, more than 10 simple repurposed drugs or three reformulation or repurposed drugs, one simple NCE, and one complex repurposed drug could potentially be launched by 2030, though the launch of an innovation-focused product would be unlikely.

### Strengths and limitations

There are three major strengths of the P2I model. The first is that we developed the model using an established portfolio methodology approach that is widely used by industry. The model assumptions on development costs, attrition rates, and cycle times for each phase were based on a very large number of data points (including data on over 25,000 product candidates), and they were “reality tested” with stakeholders out of a very large and broad group of over 130 expert stakeholders from around 80 organizations. These organizations included foundations, bilateral and multilateral development agencies, ministries of health, non-profit NGOs, organizations conducting R&D (universities, PDPs, companies, government agencies), regulatory agencies, and inter-governmental organizations. TDR’s Scientific and Technical Advisory Committee provided an additional round of expert inputs. We believe that the use of a large dataset and the extensive consultation and validation process led to a model that is based on realistic assumptions.

Second, we deliberately kept the model simple by focusing on product launches, rather than trying to include market dimensions such as anticipated price, demand, sales volumes, or profits. Investment scenarios generated by the model are based on product development costs from preclinical to launch and not on profit considerations. This approach was chosen because of the model’s focus on type II and III diseases, which disproportionately or exclusively affect people in poor countries. These patients are unlikely to be able to afford to buy new medical products themselves; it will usually be governments and external funders who purchase them.

Third, the model is highly flexible, can be used for multiple purposes, and can be modified, adapted, or built upon by users. For example, the model can be used prospectively, i.e. the user inputs the number of candidates funded in each phase and the model then estimates future costs and launches. It can be used retrospectively, i.e. the user inputs the desired number of candidates at a chosen phase, then the model determines the number of candidates needed in prior phases and forecasts associated costs and outputs. Users can change the model’s input parameters (cost, attrition rate, and cycle time per phase) to see how these changes would affect overall costs and outputs. Other archetypes can be added, as shown in an accompanying study, in which we added archetypes such as vector control products and “unprecedented vaccines” (vaccines under development for HIV, TB, or malaria, which are likely to have higher attrition rates than complex vaccines). The model can be applied to other disease areas, such as developing new technologies to combat antimicrobial resistance or to control epidemics and pandemics (e.g., the WHO R&D Blueprint for Action to Prevent Epidemic used the model to estimate financing needs to develop medical countermeasures against epidemics) ^13^.

Nevertheless, the P2I model also has a number of limitations. It is a deterministic model using single point estimates and it does not account for uncertainty or risk (it does not incorporate Monte Carlo simulations) ^14^. It assumes that all projects involving a particular archetype will have the same averaged costs, attrition rates, and cycle times per phase (e.g. all simple vaccines would have the same overall development costs). The model outputs are highly dependent on the underlying assumptions (the input parameters), which were based on aggregate data. The assumptions on attrition rates and cycle times per phase for different archetypes were based on different volumes of data—for example, data from 247 candidates for simple vaccines versus data from 14,425 candidates for innovative NCEs. The health impact estimates in the model are entirely reliant on users estimating the future expected impact, which is subject to tremendous uncertainty. Finally, the model excludes several costs, such as the costs of basic research and early discovery until lead optimization; regulatory, registration, and post-market expenditures incl. phase 4; chemistry, manufacturing and controls; good manufacturing practice; purchase of equipment; development of tools, technologies, facilities, or infrastructure; manufacture, implementation, and delivery of products.

As a consequence the expected R&D costs per launch using the P2I model lie below $1 billion (depending on archetype chosen). This is lower than other published estimates for overall drug development costs within the private pharmaceutical sector ^15^.

It is important to stress that individual product predictions are outside the scope of the model and that its real utility lies in its predictive value for modelling the impact of different funding strategies at the portfolio level.

### Next steps

The supplementary files include a User Guide and the P2I tool embedded in an Excel file, and readers are encouraged to use the tool and publish the results. An example of this adaptation is described in an accompanying paper. In that paper Young *et al.* describe how they adapted the modelling tool to create a new version, P2I V.2, in order to undertake a whole portfolio analysis of the current pipeline for those neglected diseases included in the annual G-FINDER survey of R&D financing ^16^. The model estimates it would cost about $16.3 billion to move the 538 applicable products through the pipeline, resulting in about 128 (89–160) expected product launches. Based on this analysis the model suggests there would be very few launches of complex new chemical entities and any launches of highly efficacious vaccines for HIV, TB, or malaria would be unlikely. One conclusion is that the current portfolio is not balanced across health needs.

Please note the P2I model published here in the supplementary files has been expanded to allow for modelling up to the year 2040 based on the updates described by Young *et al.*. We aim to continually refine and improve the modelling tool and ask users to provide us with their own inputs that can help us update key parameters and assumptions. We hope to catalyse users to adapt the model in ways that can increase its value, accuracy, and applications.

## Data availability

All data underlying the results are available as part of the article and no additional source data are required.

## Supplementary material

Supplementary File 1. Interview material. Zip file contain questionnaire used in the interviews and a PowerPoint describe the themes each set of questions covered.

Supplementary File 2. List of stakeholders

Supplementary File 3. Detailed cost assumptions per archetype

Supplementary File 4. Details of the health impact methodology

Supplementary File 5. The P2I tool (Microsoft Excel)

Supplementary File 6. A user’s guide to the P2I tool
