# Supplementary material for: Funding global health product R&D: the Portfolio-To-Impact Model (P2I), a new tool for modelling the impact of different research portfolios
Source: Gates Open Res. 2018 Jul 19;2:24. Originally published 2018 Apr 26. [Version 2] doi: 10.12688/gatesopenres.12816.2 (PMC6139376; doi:10.12688/gatesopenres.12816.2)
Supplement: Supplementary file 2 [file gatesopenres-2-13921-s0001.tgz › be91cbbb-592c-49b7-9080-1746eff965f5.docx]

**Supporting Information File 1. List of Stakeholders**

***Some organizations have multiple functions in the categories below, but to avoid redundancy, one category was selected depending on the expertise of the individuals contacted*

***Blue background denotes stakeholders interviewed or consulted; white denotes stakeholder contacted but not interviewed*

| Funders |  |
| --- | --- |
| Administrative Department of Science, Technology and Innovation (Colciencias), Government of Columbia | Columbia |
| African Development Bank | Nigeria |
| Asian Development Bank | Philippines |
| Bill and Melinda Gates Foundation | USA |
| Carter Center | USA |
| Department for International Development (DFID) | United Kingdom |
| Directorate-General for International Cooperation (DGIS), Netherlands Ministry of Foreign Affairs | Netherlands |
| DLR Project Management Agency, International Cooperation in Health Research | Germany |
| Dutch Ministry of Foreign Affairs (DGIS) | Netherlands |
| European Commission - Horizon 2020 | Belgium |
| Federal Ministry of Education and Research | Germany |
| Gavi, the Vaccine Alliance | Switzerland |
| German Federal Ministry for Economic Cooperation and Development (BMZ) | Germany |
| Global Health Innovative Technology Fund (GHIT) | Japan |
| Global Health Investment Fund (GHIF) | USA |
| INCLEN Trust | India |
| Indian Council of Medical Research (ICMR) | India |
| Innovative Medicines Initiative (IMI) | Netherlands |
| Irish AID, Department of Foreign Affairs and Trade | Ireland |
| KfW Development Bank | Germany |
| Medical Research Council | South Africa |
| Medical Research Council | United Kingdom |
| Multiple Myeloma Research Foundation | USA |
| National Health and Medical research Council (NHMRC) | Australia |
| National Institutes of Health | USA |
| New Development Bank BRICS (Brazil, Russia, India, China, and South Africa) | People's Republic of China |
| Novo Foundation | USA |
| Paul Allen Foundation | USA |
| Science and Technological Development Fund (STDF) | Egypt |
| Tanzania Commission for Science and Technology (COSTECH) | United Republic of Tanzania |
| The Global Fund to Fight AIDS, Tuberculosis and Malaria | Switzerland |
| United States Agency for International Development (USAID) | USA |
| Versant Ventures | USA |
| Wellcome Trust | United Kingdom |
| World Bank | USA |
| Wyss Foundation | Switzerland |
| Ministries |  |
| Federal Department of Foreign Affairs | Switzerland |
| Her Majesty's Treasury, Commercial Secretary to the Treasury, Economic, and Finance Ministry | United Kingdom |
| Ministry of Finance (former) | Nigeria |
| Ministry of Health (former) | Nigeria |
| Ministry of Health (former) | Kenya |
| Ministry of Health | Malaysia |
| Ministry of Health | Zambia |
| National Pharmaceutical Procurement Unit, Ministry of Health | Sierra Leone |
| Not-For-Profit NGOs | |
| African Federation of Public Health Associates (AFPHA) | Ethiopia |
| BIO Ventures for Global Health (BVGH) | USA |
| Deutsche Gesellschaft fur International Zusammenarbeit (GIZ) GmbH | Germany |
| European & Development Countries Clinical Trials Partnership (EDCTP) | Uganda |
| European & Development Countries Clinical Trials Partnership (former) | United Republic of Tanzania |
| European Federation of Pharmaceutical Industries and Associations (EFPIA) | Belgium |
| International Federation of Pharmaceutical Manufacturers and Associations (IFPMA) | Switzerland |
| Medecins Sans Frontieres | Switzerland |
| Medecins Sans Frontieres | France |
| Milken Institutes (FasterCures) | USA |
| Osafric Water and Energy Conservation | Kenya |
| Pharmaceutical Research and Manufacturers of America (PhRMA) | USA |
| Policy Cures | Australia |
| Organizations Conducting R&D | |
| Abbott Diagnostics | Switzerland |
| Abbott Diagnostics | USA |
| AbbVie | USA |
| Academy of Scientific Research and Technology (ASRT) | Egypt |
| AERAS | USA |
| Alere Inc | USA |
| Bavarian Nordic (BVN) | Germany |
| Bharat Biotech | India |
| Biosciences Eastern and Central Africa-International Livestock Research Institute (BecA-ILRI) | Kenya |
| Bristol-Myers Squibb | USA |
| Cairo University | Egypt |
| Department of Defense/Defense Advanced Research Projects Agency (DOD/DARPA) | USA |
| Drugs for Neglected Diseases initiative (DNDi) | Switzerland |
| Foundation for Innovative New Diagnostics (FIND) | Switzerland |
| GlaxoSmithKline (GSK) | Singapore |
| GlaxoSmithKline (GSK) | United Kingdom |
| H3 Drug Discovery Partnership | South Africa |
| Harvard Kennedy School | USA |
| Harvard School of Public Health | USA |
| Health Science Center, Peking University | People's Republic of China |
| Hilleman Laboratories | India |
| Hoffman-La Roche AG | Switzerland |
| Ifakara Health Institute | United Republic of Tanzania |
| Immunobiological Technology Institute, Bio-Manguinhos, Oswaldo Cruz Foundation | Brazil |
| Institut National de la Sante et de la Recherche Medicale (INSERM) | France |
| Instituto Nacional de Salud Publica | Mexico |
| International AIDS Vaccine Initiative (IAVI) | USA |
| Interuniversity Microelectronics Centre (IMEC) | Belgium |
| Janssen Diagnostics | Belgium |
| Janssen Diagnostics | Netherlands |
| Janssen | Belgium |
| Janssen | USA |
| Johns Hopkins University | USA |
| Johnson & Johnson | USA |
| Kenya Medical Research Institute (KEMRI) | Kenya |
| London School of Hygiene & Tropical Medicine | United Kingdom |
| Massachusetts Institute of Technology | USA |
| Medicines for Malaria Venture (MMV) | Switzerland |
| Merck & Co. Inc. | USA |
| National Institute for Pharmaceutical Research and Development (NIPRD) | Nigeria |
| Nigerian Institute of Medical Research | Nigeria |
| Noguchi Memorial Institute for Medical Research (NMIMR) | Cameroon |
| Norwegian Institute of Public Health | Norway |
| Novartis Foundation | Switzerland |
| Novartis Institute for Biomedical Research | USA |
| Novartis | Singapore |
| Novartis | Switzerland |
| Novartis (former) | Switzerland |
| Novartis | USA |
| Novavax Inc. | USA |
| Oswaldo Cruz Foundation (Fiocruz) | Brazil |
| PATH | USA |
| Roche Diagnostics | Switzerland |
| Sanon Pasteur | France |
| Sanon S.A. | France |
| Serum Institute of India | India |
| Swiss Tropical and Public Health Institute | Switzerland |
| Takeda Pharmaceutical Company | USA |
| TB Alliance | USA |
| Tropical Diseases Research Centre | Zambia |
| XOMA Ltd. | USA |
| Zhejiang University | People's Republic of China |
| Regulatory Agencies | |
| Brazilian Health Surveillance Agency (ANVISA) | Brazil |
| Centro para el Control Estatal de Medicamentos, Equipos y Dispositivos (CECMED) | Cuba |
| China Food and Drug Administration (CFDA) | People's Republic of China |
| European Medicines Agency (EMA) | United Kingdom |
| Food and Drug Administration (FDA) | Thailand |
| Food and Drug Administration (FDA) | USA |
| Korea Food and Drug Administration (KFDA) | Republic of Korea |
| Medicines Control Council | South Africa |
| National Administration of Drugs , Food, and Medical Technology (ANMAT) | Argentina |
| National Agency for Food and Drug Administration and control (NAFDAC) | Nigeria |
| Paul Ehrlich Institute, Federal Institute for Vaccines and Biomedicines (PEI) | Germany |
| Pharmaceutical and Medical Device Agency (PMDA) | Japan |
| Saudi Food and Drug Authority (SFDA) | Saudi Arabia |
| Swissmedic | Switzerland |
| Tanzania Food and Drug Administration (TFDA) | United Republic of Tanzania |
| Intergovernmental Organizations | |
| UNITAID | Switzerland |
| United Nations Children's Fund (UNICEF) | USA |
| United Nations Development Programme (UNDP) | USA |
| World Health Organization Family, Women's, and Children's Health | Switzerland |
| World Health Organization Health Systems and Innovations | Switzerland |
| World Health Organization HIV/AIDS, Tuberculosis, Malaria and Neglected Tropical Diseases | Switzerland |
| World Health Organization Noncommunicable diseases and mental health | Switzerland |
| World Health Organization Special Programme for Research and Training in Tropical Diseases (TDR) | Switzerland |
| WHO Regional Office for Africa | Congo |
| WHO Regional Office for Europe | Denmark |
| WHO Regional Office for the Americas/Pan-American Health Organization | USA |
| WHO Regional Office for the Eastern Mediterranean (EMRO) | Egypt |
| WHO South-East Asia Regional Office (SEARO) | India |
| WHO Western Pacific Regional Office (WPRO) | Philippines |
