# Supplementary material for: Funding global health product R&D: the Portfolio-To-Impact Model (P2I), a new tool for modelling the impact of different research portfolios
Source: Gates Open Res. 2018 Jul 19;2:24. Originally published 2018 Apr 26. [Version 2] doi: 10.12688/gatesopenres.12816.2 (PMC6139376; doi:10.12688/gatesopenres.12816.2)
Supplement: Supplementary file 3 [file gatesopenres-2-13921-s0002.tgz › 179442a8-922d-4e9d-9740-861d7e57d6f7.docx]

**Supporting Information File 2. Detailed Cost Assumptions per Archetype**

| **Table 1. Development cost assumptions for vaccines** | | |  |  |  |  |
| --- | --- | --- | --- | --- | --- | --- |
|  | **Phase 1** | | **Phase 2** | | **Phase 3** | |
|  | **Lower Bound** | **Upper Bound** | **Lower Bound** | **Upper Bound** | **Lower Bound** | **Upper Bound** |
| **Patient Driven Costs** |  | |  |  |  |  |
| Number of patients | 20 | 40 | 1,000 | 2,000 | 10,000 | 20,000 |
| PI Grant/patient | $3,840 | $4,800 | $2,016 | $2,592 | $2,016 | $2,592 |
| Clinical supplies | $1,728 | $2,880 | $907 | $1,555 | $907 | $1,555 |
| Monitoring | $2,880 | $4,080 | $1,512 | $2,203 | $1,512 | $2,203 |
| Labs | $960 | $1,680 | $504 | $907 | $504 | $907 |
| Data management | $768 | $1,440 | $403 | $778 | $403 | $778 |
| **Site costs** |  |  |  |  |  |  |
| Site startup cost | $24,000 | $60,000 | $24,000 | $60,000 | $24,000 | $60,000 |
| Number of sites | 1 | 3 | 1 | 6 | 18 | 24 |
| **Internal FTE costs** |  |  |  |  |  |  |
| Average annual cost/FTE | $200,000 | $200,000 | $200,000 | $200,000 | $200,000 | $200,000 |
| Clinical operations FTE | 3.52 | 3.52 | 4.69 | 4.69 | 6.25 | 6.25 |
| Contracting & legal FTE | 0.28 | 0.28 | 0.38 | 0.38 | 0.50 | 0.50 |
| Data management & statistics FTE | 0.84 | 0.84 | 1.13 | 1.13 | 1.50 | 1.50 |
| Global clinical trial management FTE | 1.13 | 1.13 | 1.50 | 1.50 | 2.00 | 2.00 |
| Global medical & regulatory affairs FTE | 0.70 | 0.70 | 0.94 | 0.94 | 1.25 | 1.25 |
| Regional clinical trial management FTE | 0.56 | 1.69 | 0.75 | 2.25 | 1.00 | 3.00 |
| Regional medical & regulatory affairs FTE | 0.70 | 1.55 | 0.94 | 2.06 | 1.25 | 2.75 |
| **Category Totals** |  |  |  |  |  |  |
| Patient Driven Costs | $203,520 | $595,200 | $5,342,400 | $16,070,400 | $53,424,000 | $160,704,000 |
| Site Driven Costs | $24,000 | $180,000 | $24,000 | $360,000 | $432,000 | $1,440,000 |
| FTE Costs | $1,546,875 | $1,940,625 | $2,062,500 | $2,587,500 | $2,750,000 | $3,450,000 |
| **Simple Vaccine Total Cost** | **$1,774,395** | **$2,715,825** | **$7,428,900** | **$19,017,900** | **$56,606,000** | **$165,594,000** |
| Incremental cost for complex vaccine | 10% | 10% | 5% | 5% | 20% | 20% |
| **Complex vaccine total cost** | **$1,951,835** | **$2,987,408** | **$7,800,345** | **$19,968,795** | **$67,927,200** | **$198,712,800** |
| **Preclinical Costs** |  |  |  |  |  |  |
| Simple (5% of total costs)* | $3,330,750 | $9,992,251 |  |  |  |  |
| Complex (10% of total costs) | $8,315,233 | $24,945,699 |  |  |  |  |
| * From: AIDS Vaccine Advocacy Coalition "Incentivizing Research and Development on Vaccines Against the Most Deadly Infectious Diseases" | | | | | | |

**Table 2. Development cost assumptions for NCEs**

|  | **Phase 1** | | **Phase 2** | | **Phase 3** | |
| --- | --- | --- | --- | --- | --- | --- |
|  | **Lower Bound** | **Upper Bound** | **Lower Bound** | **Upper Bound** | **Lower Bound** | **Upper Bound** |
| **Patient Driven Costs** |  | |  |  |  |  |
| Number of patients | 20 | 40 | 200 | 400 | 1000 | 4000 |
| PI Grant/patient | $3,840 | $4,800 | $3,360 | $4,320 | $3,360 | $4,320 |
| Clinical supplies | $1,152 | $1,920 | $1,008 | $1,728 | $1,008 | $1,728 |
| Monitoring | $2,880 | $4,080 | $2,520 | $3,672 | $2,520 | $3,672 |
| Labs | $960 | $1,680 | $840 | $1,512 | $840 | $1,512 |
| Data management | $652 | $1,200 | $588 | $1,080 | $588 | $1,080 |
| **Site costs** |  |  |  |  |  |  |
| Site startup cost | $24,000 | $60,000 | $24,000 | $60,000 | $24,000 | $60,000 |
| Number of sites | 1 | 3 | 1 | 6 | 18 | 24 |
| **Internal FTE costs** |  | |  |  |  |  |
| Average annual cost/FTE | $200,000 | $200,000 | $200,000 | $200,000 | $200,000 | $200,000 |
| Clinical operations FTE | 3.52 | 3.52 | 4.69 | 4.69 | 6.25 | 6.25 |
| Contracting & legal FTE | 0.28 | 0.28 | 0.38 | 0.38 | 0.50 | 0.50 |
| Data management & statistics FTE | 0.84 | 0.84 | 1.13 | 1.13 | 1.50 | 1.50 |
| Global clinical trial management FTE | 1.13 | 1.13 | 1.50 | 1.50 | 2.00 | 2.00 |
| Global medical & regulatory affairs FTE | 0.70 | 0.70 | 0.94 | 0.94 | 1.25 | 1.25 |
| Regional clinical trial management FTE | 0.56 | 1.69 | 0.75 | 2.25 | 1.00 | 3.00 |
| Regional medical & regulatory affairs FTE | 0.70 | 1.55 | 0.94 | 2.06 | 1.25 | 2.75 |
| **Category Totals** |  | |  |  |  |  |
| Patient Driven Costs | $190,080 | $547,200 | $1,663,200 | $4,924,800 | $8,316,000 | $49,248,000 |
| Site Driven Costs | $24,000 | $180,000 | $24,000 | $360,000 | $432,000 | $1,440,000 |
| FTE Costs | $1,546,875 | $1,940,625 | $2,062,500 | $2,587,500 | $2,750,000 | $3,450,000 |
| **Simple NCE Total Cost** | **$ 1,760,955** | **$2,667,825** | **$3,749,700** | **$7,872,300** | **$11,498,000** | **$54,138,000** |
| Incremental cost for complex vaccine | 10% | 10% | 10% | 10% | 10% | 10% |
| Phase 1 biomarker costs | $5,000,000 | $5,000,000 |  |  |  |  |
| **Complex NCE total cost** | **$6,937,051** | **$7,934,608** | **$4,124,670** | **$8,659,530** | **$12,647,800** | **$59,551,800** |
| **Simple NCE preclinical cost** | $2,500,000 | $7,500,000 |  |  |  |  |
| **Complex NCE preclinical cost** | $7,500,000 | $12,500,000 |  |  |  |  |

*NOTE: Innovative NCE costs are the midpoint between the simple NCE and complex NCE cost estimates for each phase

**Table 3. Development cost assumptions for drug repurposing**

|  | **Phase 1** | | **Phase 2** | | **Phase 3** | |
| --- | --- | --- | --- | --- | --- | --- |
|  | **Lower Bound** | **Upper Bound** | **Lower Bound** | **Upper Bound** | **Lower Bound** | **Upper Bound** |
| **Patient Driven Costs** |  | |  |  |  |  |
| Number of patients | 20 | 40 | 200 | 400 | 1,000 | 2,000 |
| PI Grant/patient | $3,840 | $4,800 | $3,360 | $4,320 | $3,360 | $4,320 |
| Clinical supplies | $1,152 | $1,920 | $1,008 | $1,728 | $1,008 | $1,728 |
| Monitoring | $2,880 | $4,080 | $2,520 | $3,672 | $2,520 | $3,672 |
| Labs | $960 | $1,680 | $840 | $1,512 | $840 | $1,512 |
| Data management | $672 | $1,200 | $588 | $1,080 | $588 | $1,080 |
| **Site costs** |  |  |  |  |  |  |
| Site startup cost | $24,000 | $60,000 | $24,000 | $60,000 | $24,000 | $60,000 |
| Number of sites | 1 | 3 | 1 | 6 | 18 | 24 |
| **Internal FTE costs** |  |  |  |  |  |  |
| Average annual cost/FTE | $200,000 | $200,000 | $200,000 | $200,000 | $200,000 | $200,000 |
| Clinical operations FTE | 3.52 | 3.52 | 4.69 | 4.69 | 6.25 | 6.25 |
| Contracting & legal FTE | 0.28 | 0.28 | 0.38 | 0.38 | 0.50 | 0.50 |
| Data management & statistics FTE | 0.84 | 0.84 | 1.13 | 1.13 | 1.50 | 1.50 |
| Global clinical trial management FTE | 1.13 | 1.13 | 1.50 | 1.50 | 2.00 | 2.00 |
| Global medical & regulatory affairs FTE | 0.70 | 0.70 | 0.94 | 0.94 | 1.25 | 1.25 |
| Regional clinical trial management FTE | 0.56 | 1.69 | 0.75 | 2.25 | 1.00 | 3.00 |
| Regional medical & regulatory affairs FTE | 0.70 | 1.55 | 0.94 | 2.06 | 1.25 | 2.75 |
| **Category Totals** |  |  |  |  |  |  |
| Patient Driven Costs | $190,080 | $547,200 | $1,663,200 | $4,924,800 | $8,316,000 | $24,624,000 |
| Site Driven Costs | $24 | $180,000 | $24,000 | $360,000 | $432,000 | $1,440,000 |
| FTE Costs | $1,546,875 | $1,940,625 | $2,062,500 | $2,587,500 | $2,750,000 | $3,450,000 |
| **Simple/Complex Repurposing Total Cost** | **$1,760,955** | **$2,667,825** | **$3,749,700** | **$7,872,300** | **$11,498,000** | **$29,514,000** |
| **Preclinical Costs** | Same as simple NCE | |  |  |  |  |

**Table 4. Development cost assumptions for biologics**

|  | **Phase 1** | | **Phase 2** | | **Phase 3** | |
| --- | --- | --- | --- | --- | --- | --- |
|  | **Lower Bound** | **Upper Bound** | **Lower Bound** | **Upper Bound** | **Lower Bound** | **Upper Bound** |
| **Patient Driven Costs** |  | |  |  |  |  |
| Number of patients | 20 | 40 | 200 | 400 | 2,000 | 4,000 |
| PI Grant/patient | $3,840 | $4,800 | $3,360 | $4,320 | $3,360 | $4,320 |
| Clinical supplies | $2,304 | $3,840 | $2,016 | $3,456 | $2,016 | $2,456 |
| Monitoring | $4,320 | $6,120 | $3,780 | $5,508 | $3,780 | $5,508 |
| Labs | $2,880 | $5,040 | $2,520 | $4,536 | $2,520 | $4,536 |
| Data management | $691 | $1,200 | $605 | $1,080 | $605 | $1,080 |
| **Site costs** |  |  |  |  |  |  |
| Site startup cost | $24,000 | $60,000 | $24,000 | $60,000 | $24,000 | $60,000 |
| Number of sites | 1 | 3 | 1 | 6 | 18 | 24 |
| **Internal FTE costs** |  | |  |  |  |  |
| Average annual cost/FTE | $200,000 | $200,000 | $200,000 | $200,000 | $200,000 | $200,000 |
| Clinical operations FTE | 3.52 | 3.52 | 4.69 | 4.69 | 6.25 | 6.25 |
| Contracting & legal FTE | 0.28 | 0.28 | 0.38 | 0.38 | 0.50 | 0.50 |
| Data management & statistics FTE | 0.84 | 0.84 | 1.13 | 1.13 | 1.50 | 1.50 |
| Global clinical trial management FTE | 1.13 | 1.13 | 1.50 | 1.50 | 2.00 | 2.00 |
| Global medical & regulatory affairs FTE | 0.70 | 0.70 | 0.94 | 0.94 | 1.25 | 1.25 |
| Regional clinical trial management FTE | 0.56 | 1.69 | 0.75 | 2.25 | 1.00 | 3.00 |
| Regional medical & regulatory affairs FTE | 0.70 | 1.55 | 0.94 | 2.06 | 1.25 | 2.75 |
| **Category Totals** |  |  |  |  |  |  |
| Patient Driven Costs | $280,704 | $840,000 | $2,456,160 | $7,560,000 | $24,561,600 | $75,600,000 |
| Site Driven Costs | $24,000 | $180,000 | $24,000 | $360,000 | $432,000 | $1,440,000 |
| FTE Costs | $1,546,875 | $1,940,625 | $2,062,500 | $2,587,500 | $2,750,000 | $3,450,000 |
| **Simple Biologic Total Cost** | **$ 1,851,579** | **$2,960,625** | **$4,542,660** | **$10,507,500** | **$27,743,600** | **$80,490,000** |
| Incremental cost for complex vaccine | 10% | 10% | 10% | 10% | 10% | 10% |
| Phase 1 biomarker costs | $5,000,000 | $5,000,000 |  |  |  |  |
| **Complex vaccine total cost** | **$2,035,737** | **$3,256,688** | **$4,996,926** | **$11,558,250** | **$30,517,960** | **$88,539,000** |
| **Preclinical Costs ^1^** |  | |  |  |  |  |
| Scaling factor relative to NCE preclinical costs^2^ | 2.16 |  |  |  |  |  |
| Biologics simple | $5,396,290 | $16,188,870 |  |  |  |  |
| Biologics complex | $16,188,870 | $26,981,450 |  |  |  |  |

1. Liao X. C. "Early Development of Antibody Therapeutics"
2. Horvath C. "Comparison of Preclinical Development Programs for Small Molecules and Large Molecules: studies, timing, materials cost" Pharmaceutical Sciences Encyclopedia 2010
