# Supplementary material for: Funding global health product R&D: the Portfolio-To-Impact Model (P2I), a new tool for modelling the impact of different research portfolios
Source: Gates Open Res. 2018 Jul 19;2:24. Originally published 2018 Apr 26. [Version 2] doi: 10.12688/gatesopenres.12816.2 (PMC6139376; doi:10.12688/gatesopenres.12816.2)
Supplement: Supplementary file 4 [file gatesopenres-2-13921-s0003.tgz › 76f8886b-0be0-4c58-af2b-d0981230c247.docx]

**Supporting Information File 3. Details of the Health Impact Methodology**

The P2I model allows users to estimate the impact of a launched product on both disability, measured in disability-adjusted life years (DALYs) averted, and mortality, measured in deaths averted. The economic value of the DALYs averted is also calculated in terms of US dollars. The user is expected to make an informed estimate of the expected reduction in disease burden and expected reduction in mortality based on project-specific characteristics and estimates including (but not limited to): impact of improved efficacy over standard of care on mortality and morbidity, coverage rates, and disease prevalence and incidence.

The health impact calculation determines the DALYs averted, associated economic value, and deaths averted for a single launched intervention, irrespective of product archetype. The 2012 disease burden (DALYs) and mortality data for each Type 3 and 2 disease are based on the Global Burden of Disease report published by the WHO in 2014 (based on data from 2012).^[[1]](#footnote-1)^ The DALYs averted metric is calculated by multiplying the 2012 LMIC disease burden by the expected reduction in disease burden (determined on a case-by-case basis by the model user). The same methodology is used for the deaths averted metric based on the users’ input.

The associated economic value of the health impact is based on a valuation of $500 per DALY averted, as used in estimates by the International Finance Facility for Immunization (IFFIm).^[[2]](#footnote-2)^ The IFFIm recognizes that the true economic value of a single DALY averted is “likely much higher,” implying that the economic value is a relatively conservative estimate of the actual economic impact.

Table 1 shows the projected DALY impact and associated economic benefits for different “expected reduction in disease burden” estimates. The figures shown represent the health impact for a single intervention launched, and would scale for multiple intervention launches.

Table 1. Health impact sensitivity table: DALYs averted and associated economic impact for a single intervention

Table 2 shows the projected reduction in mortality (deaths) for different “expected reduction in mortality” estimates. The figures shown represent the health impact for a single intervention launched, and would scale for multiple intervention launches.

Table 2. Health impact sensitivity table: deaths averted for a single intervention

The health model is heavily reliant on the user’s ability to make an informed estimate of the expected disease burden reduction and expected mortality reduction. Included below are simplified formulas for how to estimate the percentage reduction in DALYs for a treatment, vaccine, or diagnostic, which are based on a methods paper by Winfrey et al.^[[3]](#footnote-3)^

**DALYs averted**

$$\boldsymbol{R}_{\boldsymbol{DALY}}=\frac{R_{LD}YLD+R_{LL}YLL}{YLD+YLL}$$

R … per cent reduction in burden of disease due to new treatment, vaccine or diagnostic

DALY … disability-adjusted life year

YLD … Years lived with disability

YLL … Years of life lost

LD … lived with disability

LL … life lost

**Treatment or Vaccine**

$$R_{LD}=\frac{I_{LD}\times\left( C_{t}-C_{0} \right)}{1-I_{LD,0}\times C_{0}}\times AF$$

$$R_{LL}=\frac{I_{LL}\times\left( C_{t}-C_{0} \right)}{1-I_{LL,0}\times C_{0}}\times AF$$

I … effectiveness of intervention

C … coverage of intervention

AF … affected fraction of prevalent population who could be reached with treatment or vaccine

t … time of observation

0 … before introduction of intervention

**Diagnostic**

$$R_{LD}=\frac{I\times\left( C_{t}-C_{0} \right)}{1-I_{0}\times C_{0}}\times T_{LD}\times AF$$

$$R_{LL}=\frac{I\times\left( C_{t}-C_{0} \right)}{1-I_{0}\times C_{0}}\times T_{LL}\times AF$$

T … effectiveness of existing treatment

AF … affected fraction of prevalent population who could be reached with diagnostic

1. [Global Burden of Disease Study 2013 Collaborators](https://www.ncbi.nlm.nih.gov/pubmed/?term=Global%20Burden%20of%20Disease%20Study%202013%20Collaborators%5BCorporate%20Author%5D). Global, regional, and national incidence, prevalence, and years lived with disability for 301 acute and chronic diseases and injuries in 188 countries, 1990-2013: a systematic analysis for the Global Burden of Disease Study 2013. *Lancet* 2015;386:743-800. [↑](#footnote-ref-1)
2. Pearson M, Clarke J, Ward L, Grace C, Harris D, Cooper M. IFFIm evaluation: Executive summary. Geneva: Gavi, the Vaccine Alliance; 2011. [↑](#footnote-ref-2)
3. Winfrey W, McKinnon R, Stover J. Methods used in the Lives Saved Tool (LiST). BMC Public Health. 2011 Apr 13;11 Suppl 3:S32. <http://www.ncbi.nlm.nih.gov/pubmed/21501451> [↑](#footnote-ref-3)
