# Supplementary material for: Funding global health product R&D: the Portfolio-To-Impact Model (P2I), a new tool for modelling the impact of different research portfolios
Source: Gates Open Res. 2018 Jul 19;2:24. Originally published 2018 Apr 26. [Version 2] doi: 10.12688/gatesopenres.12816.2 (PMC6139376; doi:10.12688/gatesopenres.12816.2)
Supplement: Supplementary file 6 [file gatesopenres-2-13921-s0005.tgz › 11d9a021-2cbb-4f46-ad7c-06f91a5063e0.docx]

**File 5. The Portfolio-To-Impact Model (P2I) User Guide**

This user guide provides stepwise instructions on the use of the P2I model. NB to fully understand the cost assumptions and limitations of the model it should be used only after reading the main paper.

|  |  |  |  |
| --- | --- | --- | --- |
| **Model Overview** | The Portfolio-To-Impact Model (P2I Model) has been developed with a focus towards simplicity and flexibility. While the model calculations are relatively complex, the following coloured tabs should be sufficient for the most users: | | |
| **Tabs** | **Tab colour** | **Tab description** | |
| **Input sheet** | **Blue** | The user should only input known data in this tab to run the model. See below for descriptions of each input field | |
| **Output sheet** | **Orange** | This tab contains all summary tables and graphs based on the inputs from the blue "Input sheet". The "Output" tab will update automatically. **Please allow ~5 seconds for calculations to run before reviewing the output tab** | |
| **Assumptions tabs (2)** | **Green** | The model contains two green assumptions tabs (one containing assumptions for the financial model, and one for the health model). See right for additional details on the assumptions. These can be updated by inputting details in these tabs. | |

| **Using the model:** | |  |  |
| --- | --- | --- | --- |
| **Input sheet** | **There are 11 input fields to the model** | **Input field description** | **Example input** |
| **Project description** | 1. Disease | Select from a drop down menu of Type 3 and Type 2 diseases, or "other" if no Type 3/2 disease (setting this parameter only has an impact on health impact modelling) | Leishmaniasis |
|  | 2. Archetype | Select from a drop down of the 11 potential interventions (see supplement document for descriptions and examples) | NCE-simple |
| **Financial model inputs** | 3. Phase with set number of candidates | Select desired phase (preclinical, phase I, phase II, phase III, or launch) | Launch |
|  | 4. Number of candidates at phase with set number of candidates | Select number of candidates at that phase (either as an intervention objective, or as a funding decision) | 2 |
|  | 5. Model start year | Select which year to begin projects (2017 through 2030) | 2017 |
|  | 6. Model start phase | Select which phase to begin modelling (preclinical, phase I, phase II, phase III, or launch) | Preclinical |
|  | 7. Start funding at: | Select which phase to start funding (i.e., this phase will be paid for) | Preclinical |
|  | 8. Fund to: | Select which phase to fund to (i.e., all phases up to this phase will be paid for; indicated phase will not be paid for) | Launch |
|  | 9. Funding percentage (% of costs paid by fund) | Input what percentage of total costs will be paid for by the fund (e.g., to reflect in-kind contributions or co-funding from other funder) | 70% |
| **Health model inputs (OPTIONAL)** | 10. Expected impact on disease burden per launched product (% reduction of LMIC disease burden in DALYs) | Input the expected percentage reduction in low-and-middle-income-country (LMIC) disease burden for a single launched product (DALYs) | 3.00% |
|  | 11. Expected impact on mortality per launched product (% reduction of LMIC deaths) | Input the expected percentage reduction in low-and-middle-income-country (LMIC) mortality (deaths) | 1% |
| **Capacity constraints** | 12. Annual capacity constraint on number of new projects that can be started | Input the number of new projects that can enter a given phase every year (this parameter is applied per project/row, not globally) | 20.00 |

| **Assumptions Overview** | The Portfolio-To-Impact Model (P2I Model) relies on financial and health-related assumptions and results are highly sensitive to these assumptions. The pre-populated assumptions are specific to Type 3 and Type 2 diseases (as defined by the World Health Organization) or "disease of poverty" and cannot necessarily be extrapolated to other diseases or therapeutic areas. | | |
| --- | --- | --- | --- |
|  |  |  |  |
| **Tab name** | **Assumption metrics** | | **Sources** |
| **Financial modelling data sheet** | Assumptions per phase from preclinical (after lead optimization) through Phase III: --Development cost (Millions of USD) --Probability of success (percent) --Cycle time / length of phase (years) | | See supplemental document for detailed sources |
| **Health modelling data sheet** | Assumptions (baseline data) compiled for available Type 3 and Type 2 diseases (per WHO classification): --Low-and-middle-income-country (LMIC) global burden of disease (2012) --Low-and-middle-income-country (LMIC) mortality / deaths (2012) | | WHO Global Health Estimates 2014 Reports |
| **NOTE FOR DIAGNOSTICS: Inputs for the two diagnostic archetypes use the same input field and the same drop-down menu choices (i.e., preclinical, Phase I, Phase II, and Phase III. The "Selection and Validation of Markers" phase should be entered as "Preclinical". "Development" phase should be entered as "Phase I", and "Regulatory Trials beyond EUO / CE" should be entered as "Phase II"** | | | |
| **Output sheet** | **Output tables / graphs** | **Output table and graph description** | **Potential use cases** |
| **Full portfolio pipeline and cost** | Portfolio pipeline | Shows the total number of candidates (for the full portfolio) in each development phase on January 1 of each year | Review status of full portfolio pipeline projects |
|  | Total portfolio cost ($, Millions) | Shows the total expected annual costs for the full portfolio, split by development phase | Estimate total R&D costs of full portfolio |
| **Cost breakdown** | Funded portfolio cost ($, Millions) | Shows the expected annual costs paid for by the "fund"; adjusts based on inputs 7, 8, and 9 | Estimate annual funding budget needs |
|  | Portfolio costs to be paid by other funders ($, Millions) | Shows the expected annual costs NOT paid for by the fund ("Total portfolio costs" less "Funded portfolio costs") | Estimate amount of co-invested funds required from other funders |
| **Archetype breakdown** | Portfolio pipeline by archetype (Preclinical--Phase III) | Shows the total number of projects in development for each archetype on January 1 of each year | Review development status of all NCE-simple projects |
|  | Total portfolio cost by archetype ($, Millions) | Shows the annual cost of all portfolio projects in development for each archetype | Review total costs for all NCE-simple projects |
|  | Cumulative launches by archetype | Shows the cumulative number of expected launches for each archetype on January 1 of each year | Review expected number of launches for simple NCE projects by 2025 |
| **Health impact** | Health Impact metrics (based on number of launches by 2030) | Shows three health statistics for the full portfolio: 1.) reduction in LMIC disease burden, 2.) economic impact of disease burden reduction, 3.) reduction in annual mortality | Provide context to co-investment partners on potential health impact of new intervention |
