# Supplementary material for: Funding global health product R&D: the Portfolio-To-Impact Model (P2I), a new tool for modelling the impact of different research portfolios
Source: Gates Open Res. 2018 Jul 19;2:24. Originally published 2018 Apr 26. [Version 2] doi: 10.12688/gatesopenres.12816.2 (PMC6139376; doi:10.12688/gatesopenres.12816.2)
Supplement: Supplementary file 1 [file gatesopenres-2-13921-s0000.tgz › 90e2e164-7a92-4023-a2e1-ad51db289941.zip!/Interview materials/Interview notes template - v4.docx]

CONFIDENTIAL INTERVIEW NOTES

Interviewee

Click here to enter text.

Interviewer

Click here to enter text.

Location

TC

November xx, 2015

Sustainable Health Product R&D Fund: Interview notes

Background

To provide some background, there is a set of approximately 50 diseases that predominantly effect the “true” developing world. Thus, there is almost no market, only little research, the current R&D funding is small and fragmented; yet, there is a very real unmet health need. Here the World Health Assembly and the WHO came up with the concept of developing a fund to specifically support R&D in these diseases.

We from McKinsey are supporting TDR in exploring the technical and financial mechanisms that could support the development of a new health products R&D fund. We thus would like to gather input from a broad range of senior stakeholders in an anonymized way to help shape this R&D fund.

*Mention that will be treated confidentially*

Regulators

All regulators

- What are current gaps in the R&D landscape on diseases of poverty?
- How can developers ensure success from a regulatory perspective?
  - What are best practices for developers (specifically in the context of diseases of poverty)?
  - What could developers improve when interacting with regulators during the development process?
- How engaged should regulatory leaders be when assessing the potential of individual projects and its public health impact? (in a body that recommends projects to be supported through a financing mechanism)

LMIC specific regulators - additional question

- How does the financing of R&D in diseases of poverty link with your local health priorities?

R&D funding landscape

- Based on your understanding of existing R&D initiatives, which funds have been most effective at incentivizing investment in neglected diseases? Which have been least effective?
- *[Heatmap]* What has particularly worked well? *<Hypotheses to test below – not relevant, low, middle, high relevance >*
  - *Fostering partnerships between researchers and endemic countries*
  - *Professionally managed R&D with close collaboration from pre-clinical to launch and with regulatory*
  - *Robust evaluation process*
  - *Open access to and sharing of research findings, including research on early compounds*
  - *Self-sustaining replenishment mechanisms*
  - *Focus on clear and limited mission (e.g., certain disease)*
  - *Other*
- *[Heatmap]* What is lacking at the moment and what are the bottlenecks towards increasing R&D in neglected diseases? *<Hypotheses to test below – not relevant, low, middle, high relevance>*
  - *Lack of sufficient funding*
  - *Funding is sufficient but too concentrated in the top diseases*
  - *There is no unified view of global priorities hence money is too fragmented across non-priority areas*
  - *There is enough money but lack of coordination results in redundant projects and wasted effort*
  - *Pharmacos do not put their best talent towards these diseases*
  - *Other*
- Where in the current funding landscape for research of these diseases and where do you see gaps (e.g., specific diseases, specific product types)?
  - *Test for specific diseases or areas where there is not enough attention and an intervention is needed*
- *[Heatmap]* In light of this current landscape of R&D for these neglected diseases, what role could a new funding mechanism play? What would be its value proposition? *<Hypotheses to test below – not relevant, low, middle, high relevance>*
  - ***Just a body to set global priorities***
  - ***Set global priorities but also actively coordinate across private and govt. funds to ensure priority areas get the money***
  - ***Act as a “collator” of funds to actively select “projects” in priority areas and disburse funds***
  - ***Act as an independent fund-raising body to raise funds and disburse to PDPs and researchers***
  - ***Other***
- What would the benefits of such an R&D fund be?

R&D fund design and setup

- ***<If they say option 1 - global priorities>***
  - How would the funding mechanism ensure all perspectives are taken into account while framing these global priorities?
  - How would the funding mechanism ensure that these priorities are actually followed?
- ***<If they say option 2 - global priorities + active coordination>***
  - How would the funding mechanism actually do the coordination?
  - What are the key “success factors” for the funding mechanism to ensure that the various funds follow the direction being given by TDR as a coordinator role?
- ***<If they say option 3 - a collator>***
  - What distinctive advantage would this funding mechanism have over the individual funds (e.g., BMGF, Wellcome Trust)?
  - Why would the individual funds trust the “collator fund” with their money?
  - How should an optimal sustainable health fund incentivize R&D in select disease areas? Would these incentive mechanisms work even if the developed IP would have to be made publicly available? *<Hypotheses to test below – not relevant, low, middle, high relevance >*
    - Direct grants to selected projects
    - Grand challenge
    - Pull mechanisms such as market demand creation like AMC
    - Equity investment in companies
  - What type of fund disbursement mechanisms would be most suitable to different project types? What characteristics of the market / landscape would make various mechanisms more suitable?
- ***<If they say option 4 - a new fund>***
  - How do you think such a sustainable health R&D fund should finance itself? What innovative fundraising models would incentivize multiple stakeholders to contribute funds?
  - How should an optimal sustainable health fund incentivize R&D in select disease areas? Would these incentive mechanisms work even if the developed IP would have to be made publicly available? *<Hypotheses to test below – not relevant, low, middle, high relevance>*
    - Direct grants to selected projects
    - Grand challenge
    - Pull mechanisms such as market demand creation like AMC
    - Equity investment in companies
  - What type of fund disbursement mechanisms would be most suitable to different project types? What characteristics of the market / landscape would make various mechanisms more suitable?
  - How could the fund sustain itself over time? (e.g., royalty over successful assets, partial IP ownership etc., simply continue seeking donor investments)
  - Who would we have to partner with, and how, to ensure new products are actually being made available (high quality, high availability)? Would that be part of the mandate of an R&D fund?
  - Who is the right partner to host this fund? Why?

Financial model

- *[Especially for BMGF, but also others]* What methodology are you using to estimate financial costs of R&D? Can you share such a model with us?
- *[PDPs]* For modeling the costs of R&D we are building archetypes of various types. Do they seem right to you?
  - A simple vaccine would represent a vaccine where the platform has already been used to develop other vaccines, whereas a complex vaccine would require a completely novel approach
  - In terms of new chemical entity or NCE, a simple NCE would have a validated mechanism of action included, whereas a complex NCE would have a new target or mechanism of action
  - When it comes to drug repurposing, we’d speak of a simple approach if there is sufficient human data to start with Phase II trials for the repurposing, whereas in complex cases (e.g., when drug was approved for veterinary use before) even Phase I and in some cases preclinical studies are necessary
  - For biologics, we would consider simple development where the target or mechanism of action are already known, whereas complex would be where we are concerned with a new target or mechanism of action
  - Finally, for diagnostics, a simple diagnostic would be a repurposing of existing technology (e.g., changing a lab test to a kit), whereas complex diagnostic would be new technology development likely requiring clinical trials
- *[Especially for PDPs]* Can you give an estimate (rough range) of R&D costs and attrition rates per phase (Preclinical, Phase 1, Phase 2, Phase 3) for these archetypes for neglected diseases?
- In your experience, what is the best way to estimate the public health benefits / impact of a potential new treatment? E.g., how do you up-front estimate increased uptake or increased efficacy? Is there a methodology that you use specifically and could share?

Portfolio decision-making approaches

- What process and criteria do best-in-class R&D funds use to evaluate which projects should receive funding? Are there any noteworthy specific tools or methodologies you would recommend? (*e.g. impact calculator, TPPs, tracking*)
- What KPIs should be used to evaluate the overall impact of the sustainable health fund (*e.g., financial vs. nonfinancial*)? How can tracking KPIs be streamlined and standardized?
- Under what conditions would a funded project’s financial support be reduced or eliminated?

Composition & competencies needed for SWG

- What is the remit of your SWG? *(e.g. consultative-only or executive, what types of decisions)*
- How is the setup of your SWG currently? *(e.g., some permanent vs. rotating vs. ad-hoc members)*
- What are the specific competencies and skill-sets you have solved for in your SWG and why? *(Test specific competencies e.g., disease, legal, financial, business development, regulatory, ethics board)*
- How do you handle conflict of interest when appointing SWG members? What mechanisms are in place to safeguard against these?
- Given the ambitions for this R&D Fund, how would you suggest setting up an SWG for this context?

Opportunities of a TPP compendium

- If a TPP compendium would be made available, would that be valuable to you? How would this TPP compendium be used, specifically in the context of diseases of poverty? What is the biggest value it would bring, what would be your expectations?
- From your experience, how often do the TPPs tend to change and to what extend do they change in every update cycle. What would be the reasonable frequency for compendium updates?
- *[Question to entities that develop TPPs:]* Are there any TPPs available in your organization and would you be willing to share these if provided with a template? Would your organization also be willing send updated TPPs?
- Is there a template you use to develop and store TPPs (for drugs, vaccines or diagnostics), or have you heard of one, and what are its benefits?
