# Supplementary material for: Funding global health product R&D: the Portfolio-To-Impact Model (P2I), a new tool for modelling the impact of different research portfolios
Source: Gates Open Res. 2018 Jul 19;2:24. Originally published 2018 Apr 26. [Version 2] doi: 10.12688/gatesopenres.12816.2 (PMC6139376; doi:10.12688/gatesopenres.12816.2)
Supplement: Peer review history

# Peer review: Referee response for version 2

**DOI:** 10.21956/gatesopenres.13921.r26569

**Published:** 2018-07-20

**Authors:** Michael G. Head

**Affiliations:** 1 Faculty of Medicine, University of Southampton, Southampton, UK

The authors have satisfactorily revised the paper in light of comments from myself and another reviewer. I have no further corrections or suggestions.

I have read this submission. I believe that I have an appropriate level of expertise to confirm that it is of an acceptable scientific standard.

# Peer review: Referee response for version 1

**DOI:** 10.21956/gatesopenres.13883.r26545

**Published:** 2018-06-26

**Authors:** Joseph Robert Fitchett

**Affiliations:** 1 Bill & Melinda Gates Foundation, Seattle, WA, USA

The Portfolio-To-Impact (P2I) model was developed to address an important gap in the spectrum of research & development (R&D), product development, and innovation diffusion. The P2I methods and analysis by Terry et al. provide a crucial first step in efforts to more systematically evaluate product development portfolios – particularly for neglected diseases. This work should be refined by applying it “in real time” to specific diseases and to be pragmatic/salient for organizations such as the Coalition for Epidemic Preparedness Innovations (CEPI), which invest in novel vaccines to protect against three specific infections and broader platform technologies. 

1. A response number of 133 and response rate of 58% for stakeholder interviews is high and clearly helped provide valuable insights to enhance the P2I model. Could the authors clarify whether the 42% that declined were homogenous or relatively comparable in characteristics to those that agreed to contribute to the P2I consultation? The concern to mitigate here would be bias in the type of information provided by those shaping the construction of the new model. It would be helpful therefore to include the names and organizations of those that were unable to contribute to the consultations, for readers to assess salience of the model to those organizations. Looking at the supplementary figure 1, it seems a good breadth of engagement in terms of organizations was secured.
2. How did the authors identify stakeholders to engage? Considering the breadth of stakeholders approached and included, what were the unifying filters/features? Furthermore, it would be helpful for the authors to assess whether the lack of engagement from regulatory bodies (apart from the FDA and EMA) affects the findings in any way.  
3. Did the authors ask stakeholders whether any of the costs not included in the P2I model nevertheless did have an influence in decision-making when organizations review their portfolios, particularly for neglected diseases? It is conceivable that post-phase III and additional phase IV studies could play a role earlier in the product development pipeline e.g. at phase II.
4. The estimation of impact is problematic as it relies (as acknowledged in the paper) on the conservative IFFIm methodology for economic impact and on DALYs alone for health impact. As DALYs are comprised of years of lives lost and years lived with disability, which are in themselves of interest on their own depending on the archetype, would be important to couple with other health impact measures that such as disease prevalence and mortality as a measure of disease burden. In practice, a combination of 2-3 health impact measures and economic estimates could be more helpful than the single focus on DALY estimates to compare between products. This is exacerbated by the fact that comparisons are made to a single point in time (2012). Since then, the modelling behind the global burden of disease estimates methodology have significantly been updated, and focusing on a single year as time point does not factor in the most recent trends/trajectories, which would be very important for a P2I-type model to consider for decision-making.
5. An important consideration for future work is the fact that the historical data used to develop this model is likely biased, due to its (important and timely) focus on type II and type III diseases (as per the Commission on Intellectual Property Rights, Innovation, and Public Health). Data may be skewed to projects that were likely already “low hanging fruit” or most promising “quick wins”, as phrased in this paper. This could have major implications on the generalizability of the findings for future/additional investments.

Minor points

1. It would be interesting (but not essential for the purposes of this paper) to learn whether the authors were able to estimate the modelled costs by time, to help funding organizations to better calibrate their patterns of funding disbursement.
2. In Figure 3, I may have missed it but I’m not clear on what the 3M-7M represent beneath the fund titles on left hand side.
3. MIT Professor Andrew Lo’s work on R&D megafunds could be a relevant source of thinking for this group to consider.

I have read this submission. I believe that I have an appropriate level of expertise to confirm that it is of an acceptable scientific standard.

# Response

**Published:** 2018-07-10

**Authors:** Gavin Yamey

We agree the P2I modelling tool is only a first step in providing one approach to estimating the impact of an R&D portfolio.  That is our main motivation in publishing it in full and completely open access.  We encourage others to use, improve and update the tool and that is why we published two papers in parallel: one paper explaining the original P2I tool and its development and the second paper adapting the P2I to undertake the first full analysis of the neglected diseases pipeline. The original work in the TDR Report 2016 is cited by the WHO R&D Blueprint and therefore subsequently informed the thinking of the CEPI strategy. There has been some correspondence with CEPI as – at the time of writing - they are currently undertaking an analysis of the CEPI portfolio (and we are undertaking a new analysis, using the P2I tool, on diagnostics and therapeutics for the Blueprint diseases).  However, it is worth noting their costing estimate is a broader end-to-end approach encompassing basic science through to manufacture and delivery.  These elements are not covered in the P2I tool as there is much wider variability in these costs due to the choices that can be made with respect to manufacturing.  In designing P2I we felt including data with such wide variables would be the equivalent of estimating the length of an unknown piece of string so we chose to exclude them as we felt this would significantly reduce the utility of what we were trying to achieve. 

 With respect to your numbered comments we have provided our responses below.

1. With regard to the consultation we were also very grateful to the wide range of stakeholders that contributed.This is due in large part to the consultants we worked with, McKinsey & Co., who has greater access to commercial pharma data and mediated the gap between public/philanthropic and private funding.The respondents are broadly representative particularly within the product development partnerships (PDPs) which are the main mechanism currently to address the neglected diseases. Our hope is that this approach as a first step can only be improved and will provide a framework to widen the stakeholder engagement in global health priority setting and decision making.
2. The relatively limited input from regulatory bodies probably has more impact on a specific desired product profile than on a portfolio as a whole which is our main focus. As the data used in the tool is historical data that has been aggregated there is a reasonable assumption – but not tested – that the impact of regulatory requirements is already averaged across the tool and subsumed into the data.We think there might be some interesting analysis of how personalized treatments for certain conditions that would not be available to the general population might be affected more of less by regulatory requirements but this is outside the scope of this work and again would be better suited to specific cases rather than the generic analysis we present here.
3. With regard to how other costs affect decision making many of the stakeholders have commented that while this is a useful tool to gain an overview of a portfolio, the go/no-go decisions on individual projects would be very specific to the products under investigation.We highlight this in the caveats.
4. Yes we completely agree the measure of impact is quite crude.However, we decided to go with established and accepted methods to assess impact recognising their limitations.The suggestions you make on improving this measure are exactly the type of refinement and improvement we would wish to see and hope this initial piece of research will stimulate.
5. Again the point about maintaining the relevance of the data and updating the tool is completely accepted and we would welcome the opportunity to undertake this work – subject to identifying suitable resources.

# Peer review: Referee response for version 1

**DOI:** 10.21956/gatesopenres.13883.r26392

**Published:** 2018-05-02

**Authors:** Michael G. Head

**Affiliations:** 1 Faculty of Medicine, University of Southampton, Southampton, UK

This is an interesting and potentially high-impact piece of work, looking at the important topic of how funders may invest in the future with regards product development for neglected diseases. The methods are rigorous, described in detail, and their strengths and limitations well explained. Supporting data via various supplementary documents are available. Noted that the interview transcripts are not available, this is perfectly reasonable. 

 There are a few areas that could do with being revised.

 Primarily, there is no results section within the paper (though there is within the abstract). The article goes from methods straight to discussion. Whilst this is not a 'traditional' type of research, it is presented as an original research article, and there are results from the modeled scenarios that are not adequately described and would fit neatly into the results section.

 The abstract also needs some rewriting, a few points below, but overall it could be a better shop window for the work. There will be potential readers who want to scan the abstract to get the thrust of the findings and "what's new", at the moment it's a little unclear. 

 For these reasons, I think the paper should certainly be indexed, but for now I've categorized as 'approved with reservations'.

 A few more specific points:

- Title - doesn't have a study descriptor in it. As it's written as an original research article, then ideally needs to clearly state e.g. 'a modelling exercise' or something that tells the reader the type of investigation contained therein. Given there are modelling and stakeholder interview components, then 'a mixed-methods approach' or similar might also be appropriate
- Methods, abstract - 'Parexel's R&D cost sourcebook'. Interested readers won't necessarily know what this is, suggest add a few explanatory words to demystify it a little.
- Results, abstract - requires complete revision (what's currently written there aren't results). 
- Conclusions, abstract - the first sentence, 'this article describes...'. Delete this. Not really a conclusion per se, and I'd at this point be rather surprised if the rest of the article didn't tell me more about the modelling tool. I think give the conclusions some further attention once the results section is revised.
- Introduction - 
  - End of first paragraph, suggest revise to ''public or philanthropic sources'
  - Second paragraph - Worth half a sentence to say what TDR is/does, for the uninitiated? 
  - Further down, below the type I - III decriptions. Reference initially to 'studies', then to 'study'
  - Top of column 2, 'time period 2017-2040'. Briefly justify why 2040, why not 2030, or 2050?
- Methods
  - Page 6, column 1, 'for both cases data was sourced from PDP stakeholder...'. 
  'both cases' - is this referring to the assumptions of success probability and cycle times? Suggest rewording from 'cases' to 'scenarios' or 'assumptions' or similar
- Figure 3. Seven scenarios in table 6, but only five shown in figure 3?
- Discussion -
  - What did happen to the results section?
  - Page 10, column 2, "seven different scenarios were modelled..."; this and some ensuing text is results rather than discussion
  - Strengths and limitations, the list of organisations covers would be better in methods rather than a discussion section
  - If enthusiasm and word count allows, then there could be a fuller discussion of how the relevant stakeholders make priorities at the moment, the large number of stakeholders with limited communication between them even in the same sectors, what data is available to them to inform their strategy/intelligence (e.g. ResIn, gfinder etc). And then you have a natural point to move the discussion onto 'therefore, this tool can help in these specific areas...'

I have read this submission. I believe that I have an appropriate level of expertise to confirm that it is of an acceptable scientific standard, however I have significant reservations, as outlined above.

# Response

**Published:** 2018-07-10

**Authors:** Gavin Yamey

Again many thanks for the carefully considered comment and editorial suggestions.  In response:

 The mix-up of results with methods has been resolved with a specific results section.  Much of the detailed discussion and context for this study has already been published in the TDR Report 2016 so we accept that the results in this paper were probably underreported and we have attempted to redress that.

 You highlight a number of areas in the text requiring changes and improvement and for the most part we have made those changes.  The confusion over the numbers of financial mechanism scenarios we developed in the TDR Report 2016 (7) and the use of the P2I tool in generating the impact of different fund sizes (5) has been resolved.  Figure 3 has also been updated and replaced.

 With regard to a wider discussion about how the impact of product development for global health might be improved with greater communication and subsequent coordination between all the actors we agree but felt we wanted to keep this paper focused on the tool and its use rather than the implications for its use.  Again there is more context in the TDR Report 2016 and the parallel paper by Young *et al* analysing the neglected diseases pipeline.  The lead author, R F Terry, has published a number of papers on how greater transparency in R&D funding would be the first step towards improving coordination and this led in part to the creation of the WHO Global Health R&D Observatory (see reference below).

 Again we would hope that by creating this tool, whilst recognising its limitations, we will stimulate and support greater efforts at reaching the goal of improved coordination between the wide range of stakeholders.

**References:**

 Creating a Global Observatory for Health R&D. Robert F. Terry, José F. Salm Jr., Claudia Nannei, Christopher Dye. Science 12 Sep 2014 : 1302-1304 Publisher Full Text
